# Association of Plasma Alzheimer’s Disease Biomarkers with White Matter Hyperintensity Volume in Diverse Populations: A HABS‐HD Study

**DOI:** 10.1002/alz.093467

**Published:** 2025-01-09

**Authors:** Antoine R Trammell, James J. Lah, Felicia Goldstein, Monica W Parker, Chadwick M Hales, Crystal P Davis, Cornelya D Dorbin, Sabria Saleh, Elizabeth Murphy‐Tchivzhel, Alecia Michella Rose, Sid E. O'Bryant

**Affiliations:** ^1^ Emory University Goizueta Alzheimer's Disease Research Center, Atlanta, GA USA; ^2^ Emory University, Atlanta, GA USA; ^3^ Emory University School of Medicine, Atlanta, GA USA; ^4^ University of North Texas Health Science Center, Fort Worth, TX USA

## Abstract

**Background:**

Older African American (AA) and Hispanic American (HA) adults have a higher risk for Alzheimer’s disease (AD) than older non‐Hispanic white (NHW) adults. Cerebrovascular disease reflected by white matter hyperintensity (WMH) lesions on magnetic resonance imaging (MRI) may influence ADRD risk in these groups. Amyloid and tau PET data from studies of mostly NHW participants show a relationship between AD pathology and WMH lesions. Using a diverse cohort, this study investigates relationships between plasma AD biomarkers and WMH volume.

**Method:**

We analyzed plasma AD biomarkers, WMH volume, and self‐reported participant race (NHW, HA, AA) obtained during baseline visits for the Health and Aging Brain Study: Health Disparities. Plasma AD biomarkers included amyloid‐beta40 (Aβ40), amyloid‐beta42 (Aβ42), total tau (T‐tau), phosphorylated tau181 (P‐tau181), and neurofilament light (NfL) from single‐molecule enzyme‐linked immunosorbent assays. From self‐reported race and baseline global Clinical Dementia Rating scores (normal=0, impaired≥0.5), we created race/cognitive groups (white/normal, white/impaired, AA/normal, AA/impaired, HA/normal, HA/impaired). Using ANOVA and chi‐square, we compared baseline characteristics, plasma AD biomarkers, and WMH volume in the groups. We tested associations between plasma AD biomarkers and WMH volume with linear regression adjusted for age, education, and sex.

**Result:**

Data from 3,035 participants was analyzed (mean age: 65.2; female: 62.3%; impaired: 20.1%). AA persons were younger (NHW: 68.6, AA: 62.9, HA: 62.3. p<.0001) and had the highest WMH volume (NHW: 4.16, AA: 18.4, HA: 3.18, p<.0001). AA and HA participants had the lowest Aβ42 (NHW:10.9, AA:10.2, HA:10.1, p<.0001), while HA persons had the lowest Aβ40 (NHW: 223, AA: 220, HA: 207, p=0.0003). NHW persons had the highest P‐tau181 (NHW: 2.38, AA: 1.95, HA: 1.92, p<.0001) and NfL (NHW:19.1, AA: 12.3, HA: 17.1, p<.0001). Regression showed higher WMH volume was associated with higher Aβ40 (β=.02, p=.02), P‐tau181 (β=.29, p=.01), and T‐tau (β=1.55, p=.0002) in the HA/impaired group.

**Conclusion:**

Our results show associations between plasma AD biomarkers and WMH volume in cognitively impaired HA adults who also have low WMH burden compared to other groups. Further studies to discern potential mechanisms and the potential diagnostic prognostic utility of plasma AD biomarkers on AD risk are needed.

